# Endovascular Management of a Refractory Pseudoaneurysm of the Sternocleidomastoid Artery Caused by Attempted Internal Jugular Central Line Placement with Long-Term Follow-Up: A Case Report and Review

**DOI:** 10.1155/2018/8324908

**Published:** 2018-10-10

**Authors:** Yassmeen Abdel-Aty, Michael P. Bellew

**Affiliations:** ^1^Mayo Clinic Arizona, Department of Otolaryngology Head and Neck Surgery, 5777 E Mayo Blvd, Phoenix, AZ 85054, USA; ^2^Department of Medical Education, Assistant Professor of Neurosurgery, UCF College of Medicine, 6850 Lake Nona Blvd, Orlando, FL 32827, USA; ^3^Florida Hospital Orlando, 601 East Rollins Street, Orlando, FL 32803, USA

## Abstract

**Introduction:**

This case report shows successful treatment of a refractory sternocleidomastoid branch of the superior thyroid artery (SBSTA) pseudoaneurysm using endovascular glue embolization in a patient who refused surgery.

**Case Presentation:**

A 63-year-old female with multiple comorbidities presented with a firm 7 cm tender mass located in the right neck. Ultrasound showed pseudoaneurysm and a 7 × 3.3 × 4 cm multilobular hematoma in the location of the previous central line. CTA showed a corresponding heterogeneous mass. Serial imaging demonstrated enlargement over 2 weeks. Angiogram showed contrast blush off of the SBSTA.

**Management and Outcome:**

SBSTA was embolized using glue. Repeat angiogram showed embolization and no contrast blush. One month later, the mass was no longer pulsatile but present on physical exam. CTA showed decreased size. 8 months later, her neck was soft without mass.

**Discussion:**

Pseudoaneurysms of the external carotid artery are rare and usually due to trauma. Pseudoaneurysms after central line placement are documented, but most are complications of femoral central lines. A handful of cases of superior thyroid artery pseudoaneurysms due to several etiologies are reported, but none involving the SBSTA. Therapeutic options include surveillance, compression, thrombin injection, embolization, and surgery. Endovascular management offers an alternative for patients unwilling or unable to undergo open surgery.

## 1. Introduction

Pseudoaneurysms are a tear though all layers of an artery allowing blood to leak into the surrounding tissue causing a mass that often gradually expands due to its communication with the arterial lumen. Signs and symptoms of pseudoaneurysms in the neck can include a pulsating mass which is the most common presentation, craniocervical pain, dysphagia, hoarseness, and neurologic deficits due to cranial nerve palsy or stroke.

A pseudoaneurysm of the external carotid artery (ECA) is very rare with an incidence of 0.07% and mortality of 30% [1]. It usually occurs if the trunk or branches of this vessel are damaged. This is most likely due to trauma of the neck but can also be due to iatrogenic complications such as surgery in this area and, to a lesser degree, central line placement [2].

Around 7 million central venous catheters are placed yearly in the United States. It is a relatively safe procedure, but complications do occur. The reported incidence of serious complications varies from 0.4% to 9.9%. Accidental arterial puncture occurs in approximately 5% of all central venous catheter placements and is more likely in internal jugular vein catheterization. This can lead to arterial dissection, arteriovenous fistula formation, hematoma, pseudoaneurysms, and stroke [3]. Pseudoaneurysm formation after central line placement has been well documented in the literature but most of these cases are complications of femoral central line attempts [3].

Although cases of ECA pseudoaneurysms have been reported as well as a handful of cases of superior thyroid artery pseudoaneurysm, no cases of a sternocleidomastoid artery pseudoaneurysm have previously been reported to our knowledge. The sternocleidomastoid artery is one of the five branches of the superior thyroid artery which is a branch of the external carotid artery. It can be identified because it is the only branch of the superior thyroid artery that courses laterally [4]. This case study details a management option for aneurysms refractory to standard treatment methods as well as the time course of hematoma resolution during midterm (1 month)/long-term (8 month) follow-up.

## 2. Case Report

A 63-year-old woman presented with a firm 7 cm multilobular tender mass located in the region of the right sternocleidomastoid directly above the clavicle. She had multiple comorbidities including renal failure, systemic lupus erythematosus, and protein S deficiency, a history of transient ischemic attack, deep vein thrombosis, and pulmonary embolism, and longstanding malnourishment requiring jejunostomy tube placement. The firm mass was identified 1 month after an unsuccessful attempt at placing a right internal jugular vein central line. When the central line was originally placed, she developed pain and swelling of her neck. The swelling was pulsatile at that time and enlarging. She was offered surgery as the standard of care for her condition which she refused because she did not want to undergo an invasive procedure and was aware of the risks involved with holding her anticoagulant medication. She had two thrombin injections in the mass since without success. She complained of right neck pain. She was not having any difficulty breathing and denied shortness of breath.

**Figure 1:**
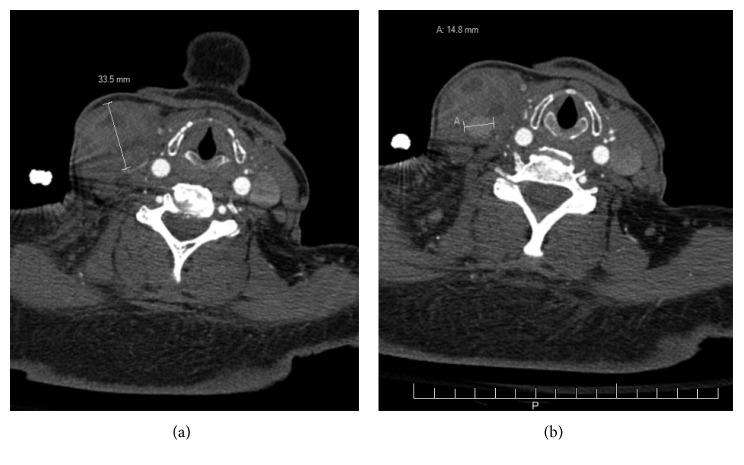
CT angiography of neck: (a) right neck mass measured and (b) right neck pseudoaneurysm measured.

Soft tissue ultrasound after the two thrombin injections showed pseudoaneurysm and a 7 × 3.3 × 4 cm multilobular hematoma at the base of the right neck. CTA of the neck showed a corresponding heterogeneous mass. The pseudoaneurysm was seen measuring 1.5 cm at the internal margin of the hematoma. Serial imaging with CTA demonstrated enlargement over a 2-week interval (Figure 1).

**Figure 2:**
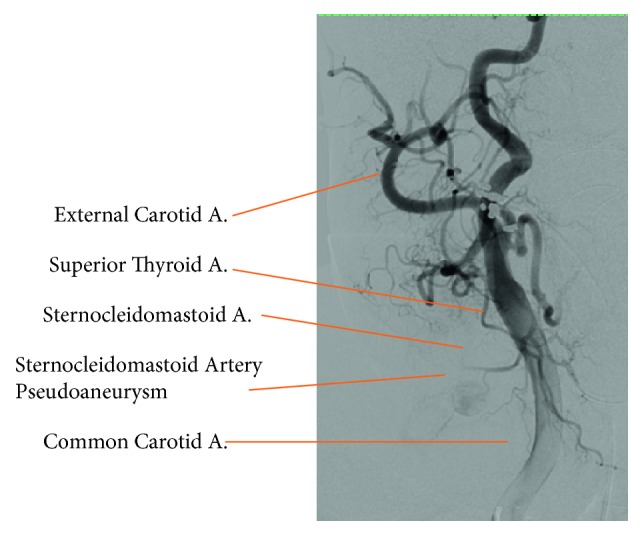
Angiogram of right common carotid and its branches. This figure depicts the pseudoaneurysm of right sternocleidomastoid artery prior to embolization as well as shows vascular anatomy rarely identified in the operative setting.

Angiogram showed blush of contrast coming off of the sternocleidomastoid branch of the superior thyroid artery identifying the location of the pseudoaneurysm (Figure 2). This branch was embolized using glue. Repeat angiogram showed successful embolization and no contrast blush.

**Figure 3:**
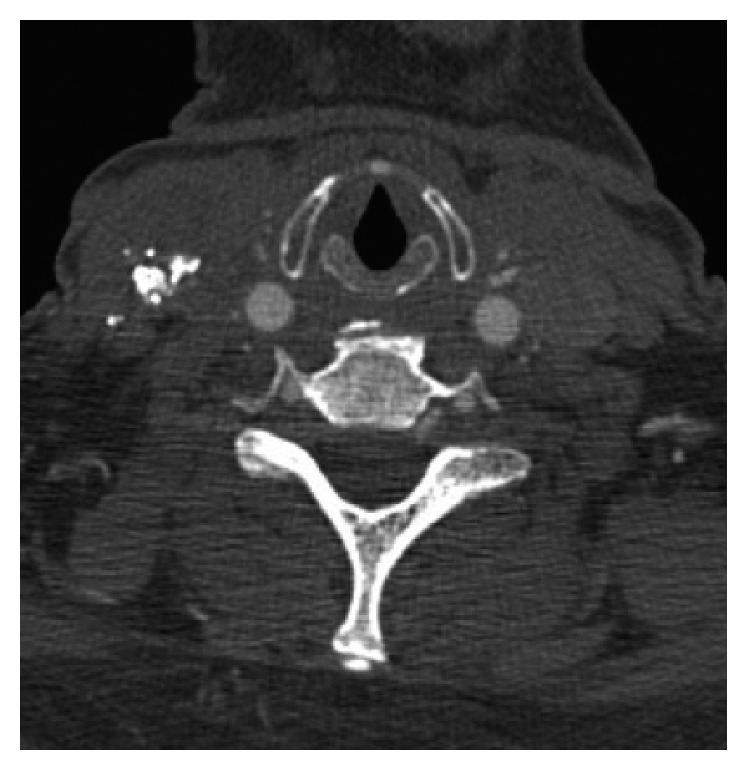
CT angiography neck: right neck mass on 1 month follow-up status after right pseudoaneurysm embolized with glue. These can be compared to the preintervention CT in Figure 1 showing that the neck mass has improved.

On follow-up one month later, the mass was no longer pulsatile but still present on physical exam. CTA of the neck showed a decrease in size from the previous CTA (Figure 3).

On follow-up 8 months later, she was found to have a soft neck with no mass on physical exam.

## 3. Discussion

Diagnostic imaging can be used when history and physical examination point to possible pseudoaneurysm. Doppler ultrasound is a very helpful and benign test that can reveal a pseudoaneurysm as a spindle shape or sac-like dilation. It can show turbulent flow and vessel dilation with a 95% accuracy for pseudoaneurysm. It is less effective for deeper lesions which may be better seen on contrast-enhanced CT or angiography. Using angiography for diagnostic purposes is not first line since it is a more invasive procedure but can be utilized when it is also intended as a therapeutic option after other imaging has been performed [2].

Therapeutic options include surgical resections, conservative surveillance, compression, thrombin injection, and catheter-based embolization. The gold standard treatment of pseudoaneurysm is surgical repair [3]. As with any surgery in the neck, surgical repair does not go without risks. Studies have shown that many iatrogenic pseudoaneurysms will heal spontaneously and as many as 89% of them will resolve spontaneously [2]. This suggests that if the pseudoaneurysm is not causing any major complications it is acceptable to watch it conservatively. However, it must be watched very closely because hemorrhage into the neck or compression of vital structures in the neck could be devastating.

Ultrasound-guided thrombin injection relies on the formation of a clot within the pseudoaneurysm to exclude it from the circulation. Case reports have shown success of ultrasound-guided thrombin injection [1]. This ensures that thrombin is injected directly into the pseudoaneurysm. There is also risk of incomplete occlusion or recannulization which may require additional therapy [5]. Since our patient refused surgery, trying thrombin injections was reasonable but unsuccessful.

A study done at the Ninth People's Hospital, Shanghai Jiao Tong University concluded coil embolization by angiography of pseudoaneurysms coming off the external carotid artery is a useful alternative to standard surgical repair and avoids morbidity associated with performing surgery in the face and neck area [2]. Many case reports have also documented success of endovascular embolization of pseudoaneurysms of the ECA and its branches [6, 7]. In our case, we have shown that glue can also be used to successfully resolve a pseudoaneurysm endovascularly.

## 4. Conclusion

Pseudoaneurysm should be included in differential for neck mass. A pseudoaneurysm due to central venous catheter placement is a very rare occurrence. Although cases of external carotid artery pseudoaneurysms have been reported, most are due to trauma. A handful of cases of superior thyroid artery pseudoaneurysms have been reported but no cases of sternocleidomastoid artery, a branch of the superior thyroid artery, pseudoaneurysms have previously been reported to our knowledge. This case study shows a successful resolution of a sternocleidomastoid artery pseudoaneurysm using glue endovascularly. Other treatments that were attempted were conservative management and thrombin injections, but these failed to resolve this woman's symptoms. While conservative management and compression may be good therapies to try initially in patients who are not in imminent danger, surgery and endovascular treatment are better therapies in pseudoaneurysms that are resistant to treatment or in unstable patients. Although surgery is the gold standard, endovascular treatment provides a less invasive option when the patient is not a good candidate for surgery or refuses surgical intervention. Thrombin injections may also be attempted but are not as safe without concurrent endovascular cerebral protection.
